# A Leakage-Free Survival-Modelling Benchmark for Hepatocellular Carcinoma Recurrence After Liver Transplantation: Nested Cross-Validation Against the Milan Criteria

**DOI:** 10.3390/bioengineering13080951

**Published:** 2026-08-21

**Authors:** Sami Akbulut, Cemil Colak, Emek Guldogan

**Affiliations:** 1 Department of Surgery and Liver Transplantation, Faculty of Medicine, Inonu University, 44280 Malatya, Türkiye; 2 Department of Biostatistics and Medical Informatics, Faculty of Medicine, Inonu University, 44280 Malatya, Türkiye; cemilcolak@yahoo.com (C.C.); emek.guldogan@inonu.edu.tr (E.G.)

**Keywords:** hepatocellular carcinoma, liver transplantation, recurrence-free survival, survival analysis, data leakage, nested cross-validation, clinical prediction model, decision-curve analysis

## Abstract

**Background**: Predicting recurrence after liver transplantation (LT) for hepatocellular carcinoma (HCC) remains important for post-transplant risk stratification and surveillance planning. The Milan criteria discriminate only moderately and some machine-learning re-analyses report overly optimistic results because of information leakage. **Aim**: The current study aimed to re-evaluate a previously published transplant cohort under a leakage-free survival-analysis framework and to benchmark post-transplant, explant-informed survival learners against the Milan criteria as a fixed pre-transplant reference. We hypothesised moderate rather than near-perfect discrimination, similar performance across learners of differing complexity, and better discrimination than the Milan criteria. **Methods**: This secondary analysis included 356 patients with HCC who underwent LT. The primary endpoint was recurrence-free survival, analysed from the observed event indicator and follow-up time rather than from a derived risk label. Seven survival learners were benchmarked with repeated nested cross-validation, using three repeats of a five-fold outer loop with a three-fold inner tuning loop. All data-dependent preprocessing, including robust multivariable outlier handling and imputation, was fitted within training folds only. Performance was assessed by the concordance indices of Harrell and Uno, the time-dependent area under the curve, the integrated Brier score, calibration, decision-curve analysis and descriptive competing-risk assessment. **Results:** Recurrence developed in 183 of the 356 patients over a median follow-up of 52 months. Discrimination was moderate rather than near-perfect and similar across learners; the random survival forest ranked highest and the Elastic-Net Cox model performed comparably. All learners showed higher descriptive concordance than the Milan criteria, and dependency-corrected comparisons supported higher concordance for the full-feature Cox model than for the Milan criteria, whereas the random survival forest and Cox did not differ materially. Out-of-fold calibration of the Elastic-Net Cox model at 36 months was acceptable, decision-curve analysis indicated positive net benefit across clinically relevant thresholds, and tumour size and alpha-fetoprotein were the leading contributors to prediction. Findings were stable in ablation and threshold-sensitivity analyses. **Conclusions**: Leakage-free survival modelling gave moderate but internally validated prediction of post-transplant recurrence and higher concordance than the Milan criteria in this cohort, supporting the stated hypotheses. Careful study design may matter more than architectural complexity in this setting, and leakage-free survival analysis is a practical standard for prognostic modelling in transplant oncology.

## 1. Introduction

Hepatocellular carcinoma (HCC) remains one of the leading causes of cancer-related death worldwide. In carefully selected patients with cirrhosis and limited tumour burden, liver transplantation (LT) offers a major oncologic advantage because it removes both the tumour and the diseased liver. However, this benefit is reduced by post-transplant recurrence, which remains closely linked to poor long-term survival. For this reason, the balance between transplant benefit and recurrence risk is a central issue in the selection of patients for LT [1,2,3].

The modern approach to LT for HCC was largely shaped by the Milan criteria [4]. These criteria showed that strict morphological selection could achieve acceptable post-transplant outcomes and they still serve as the main benchmark for newer prediction tools. At the same time, their limitations are well known. Milan is based mainly on tumour size and number, and therefore reflects tumour burden more than tumour biology. In daily practice, some patients within Milan still develop recurrence, whereas some patients beyond Milan have good long-term outcomes. This gap has led to many attempts to improve risk assessment beyond a simple anatomic threshold [2,3].

Over time, it became clear that morphology alone is not enough for individual risk prediction. As a result, many studies have examined biological and dynamic markers of tumour behaviour, including alpha-fetoprotein, response to locoregional treatment, inflammatory markers, tumour burden scores, and imaging features associated with aggressive disease. This led to the development of several composite models such as the AFP model, Metroticket 2.0, MORAL, HALT-HCC, and RETREAT. More recent data-driven tools, including RELAPSE and TRAIN-AI, follow the same overall direction by combining morphological, biological, and treatment-response variables in larger datasets. The shared aim of these approaches is to estimate recurrence risk in a more individual and biologically informed way rather than to classify patients by tumour size and number alone [2,3,5,6].

An important point, however, is that these models are not built for exactly the same clinical purpose. Some are designed for pre-transplant candidate selection and use only variables available before surgery. Others include explant pathology or other postoperative findings and are therefore more relevant to post-transplant surveillance and risk stratification than to organ-allocation decisions. This difference matters. A model that includes microvascular invasion, histological grade, or other explant findings may perform better, but it is solving an easier problem because it uses information that was not available when the transplant decision was made. For that reason, stronger post-transplant performance does not necessarily mean better support for pre-transplant patient selection [7,8,9].

Against this background, artificial intelligence (AI), machine learning (ML), and deep learning (DL) have attracted increasing interest in this field. The rationale is that recurrence after LT is likely influenced by complex relations among tumour burden, biomarkers, treatment response, pathology, and imaging findings, and these relations may not be fully captured by traditional regression models. As a result, a growing literature has explored histopathology-based DL, radiomics, multimodal imaging models, and structured clinical ML pipelines for recurrence prediction after LT. At the same time, broader reviews suggest that recurrence modelling is now part of a wider expansion of AI applications across the transplant pathway rather than an isolated topic [5,7,8,10,11,12,13,14,15,16,17,18].

Still, the reported results vary widely. This is not surprising because these studies differ in several important ways. They do not all predict the same endpoint. Some focus on early recurrence within a fixed time window, whereas others analyse recurrence-free survival over time. Some use binary classification and area-under-the-curve metrics, whereas others use time-to-event methods and concordance measures. Some are intended for pre-transplant decision-making, whereas others include postoperative or explant variables. In addition, many imaging-based studies are retrospective, single-centre, and based on relatively small cohorts, and many use random train-test splits rather than truly independent external validation. Because of this heterogeneity, headline performance figures cannot be compared directly across studies [8,12,13,15,16,17,18].

This issue is clinically important. For transplant candidate selection, the key question is not whether a model can separate very high-risk from very low-risk patients in retrospect using rich postoperative information, but whether it can provide reliable estimates using data that are truly available at the time of decision-making. Recent studies illustrate this point. In multicentre and registry-based analyses, pre-transplant models usually show moderate discrimination, whereas post-transplant models perform better after clinicopathologic variables are added. External validation studies support the same message. For example, the TRIUMPH model reached a c-index of about 0.71 across six international centres, which is useful but still far from near-perfect prediction. Radiomics and multimodal imaging models may show higher values, especially for early recurrence, but they often address a different endpoint and may face practical problems with generalisability across scanners, centres, and segmentation methods [8,9,15,16,17,19].

Another important problem is overoptimistic performance estimates caused by the study design. In prognostic research, strong performance is meaningful only when the outcome definition, preprocessing, model tuning, and validation strategy reflect the way the model would actually be used. This is especially important when the prediction target is simplified or derived from variables that later reappear in the model. In the previously published cohort re-analysed here, a pycox-based approach was followed by TabNet classification after patients were grouped as high or low risk according to tumour size, grade, and number. Although the reported accuracy was high, classification of a derived risk label is not the same as direct modelling of the observed recurrence event and follow-up time. In general, classifying a constructed label is easier than modelling censored time-to-event recurrence, and results from that design should therefore be interpreted with caution [13,20].

Against this background, the aim of the present study is not to introduce a new architecture, but to establish what level of performance is honestly attainable in this post-transplant recurrence-risk modelling setting once leakage is removed by design. We therefore model the observed recurrence-free survival event and time directly, fit all data-dependent preprocessing steps within the training folds, and use nested cross-validation to keep model selection separate from final evaluation. In this setting, survival modelling, fold-specific preprocessing, and nested cross-validation are not technical refinements; they are necessary safeguards to account for censoring, avoid leakage, and obtain a more credible estimate of out-of-sample performance. The central question is deliberately conservative: how well can post-transplant recurrence be predicted when the analysis is held to a leakage-free standard, and how does that honest performance compare with the Milan criteria as a fixed clinical benchmark rather than as an equivalent pre-transplant model? On this basis we tested three prespecified hypotheses: First, once leakage is removed by design, the attainable discrimination for post-transplant recurrence in this cohort would be moderate rather than near-perfect, and clearly below the accuracy previously reported for the derived-risk-label classification of the same data. Second, survival learners of markedly different complexity, from penalised Cox regression to tree-based ensembles, would not differ materially from one another. Third, these learners would nevertheless show higher concordance than the Milan criteria applied as a fixed morphological reference. Whether each hypothesis was confirmed or refuted is stated explicitly in the Discussion and in the Conclusions.

## 2. Material and Methods

### 2.1. Data Source and Study Cohort

This secondary analysis used a previously published, de-identified cohort of 356 patients with HCC who underwent LT at a single tertiary centre [13]. Each row represented one patient, and no repeated measurements were included. Patients were therefore treated as statistically independent, grouped cross-validation was not required, and stratification by event status was considered sufficient [20]. Because the analysis was based entirely on previously published anonymised data, no new patient contact or identifiable information was involved. During the data audit, the maximum α-fetoprotein (AFP) value in the public dataset was approximately 275,924 ng/mL, which was about one order of magnitude higher than the upper value reported in the original baseline table. These values were retained. Their potential influence was addressed with the robust multivariable procedure described below and was examined again in the sensitivity analysis.

### 2.2. Outcomes and Predictors

The primary outcome was recurrence-free survival (RFS), defined by the observed event indicator and event time provided in the cohort. Overall survival was not modelled as a separate outcome, because the supplied vital-status indicator is not separable from recurrence in this file (see the cross-tabulation below); the analysis therefore reports the recurrence-free survival endpoint only. In contrast to the original analysis, no derived risk label was used as the prediction target. Using the observed event-time outcome directly avoids target-construction leakage at the source [20]. Cross-tabulation of the supplied data showed 183 RFS events and 167 death indicators. All 167 deaths occurred among RFS = 1 records; no death indicator occurred among the 173 RFS = 0 records. Death without recurrence was therefore not separately observable, although the file cannot distinguish endpoint incorporation from true absence. In the supplied file the vital-status field is coded 1 = death and 0 = alive at last contact. This direction was verified within the file: all 167 death records have a survival time strictly greater than the corresponding recurrence time, whereas the 173 recurrence-free records are censored with survival time equal to recurrence time and a minimum follow-up of 48 months.

A total of 25 candidate predictors were evaluated. These comprised demographic characteristics (age and sex), viral hepatitis status (HBV and HCV infection), serum tumour markers (AFP and CA 19-9), background liver disease (cirrhosis), ABO blood group, previous abdominal surgical history (history of abdominal surgery, previous laparotomy, and splenectomy), pre-transplant treatments (previous liver resection, transarterial chemoembolization, and radiofrequency ablation), intraoperative variables (estimated blood loss and blood transfusion), and tumour-related pathological characteristics (tumour number, maximum tumour diameter, satellite lesions, histological grade, microvascular invasion, portal vein tumour thrombus [PVTT], PVTT location, bile duct tumour thrombus, and inferior vena cava invasion). Ordinal variables, including histological grade, were encoded numerically using a prespecified fixed mapping. Because the mapping was defined independently of the study data and did not use information from other patients or outcome values, its application before cross-validation was not considered a source of data leakage [20].

### 2.3. Leakage-Free Preprocessing and Robust Outlier Handling

All data-dependent preprocessing steps were placed within a single estimator pipeline and re-fitted from the beginning in the training partition of each fold. In this way, no preprocessing parameter was informed by held-out data. Continuous predictors were screened for multivariable outliers using a robust Mahalanobis distance based on the Minimum Covariance Determinant estimator, with a chi-squared threshold. In flagged records, only the cells contributing most strongly to the deviation were set to missing on the basis of a median absolute deviation robust z-score. These values were then re-estimated by multivariable iterative imputation, followed by log transformation and standardisation. The robust centre, scatter matrix, chi-squared threshold, and imputation model were learned only from the training fold and then applied unchanged to the corresponding test fold. This approach mirrors the clinical setting in which a model trained earlier is applied to a new patient and helps prevent leakage that can arise when preprocessing is fitted on the full dataset before data splitting [20].

### 2.4. Survival Learners and Hyperparameter Grids

Seven survival learners with different modelling assumptions were benchmarked using the scikit-survival library [21]: a penalised Cox model with an Elastic-Net penalty (CoxNet), a ridge-penalised Cox model, a random survival forest [22], an extremely randomised survival-tree ensemble, gradient-boosted survival trees, a component-wise linear gradient-boosting model, and a ranking survival support vector machine. The Cox models provided interpretable regularised linear baselines [23]. The tree-based models were included to capture non-linear relations and interactions without prespecifying them. The ranking survival support vector machine optimised concordance directly, but because it did not produce a full survival function, it contributed only to concordance-based evaluation. As a non-trainable clinical benchmark, the Milan criteria were applied as a fixed rule in every outer test fold so that comparisons were made on the same patients. The rule was operationalised from the tumour-number and maximum-diameter fields as a single lesion of 5 cm or less, or up to three lesions each of 3 cm or less; patients outside these limits were assigned the higher-risk score. Because the supplied dataset records tumour number as single versus multiple rather than as an exact count, the three-lesion element of the rule could not be applied literally; patients with multiple lesions and a maximum diameter of 3 cm or less were therefore classified as within the criteria. For the additional reproducibility analysis, ridge Cox alpha was searched over [0.1, 1.0, 10.0, 100.0] using inner-fold Harrell concordance; random survival forest used 200 trees, minimum leaf size 8, and square-root feature sampling. Every candidate value was evaluated in every inner fold, so this search comprised 4 candidates × 3 inner folds × 15 outer folds = 180 model fits, and alpha = 100 was selected in all 15 outer folds. The complete search space of every learner, including the tuned parameter names, the exact candidate values, the number of candidate combinations and the resulting number of model fits, is reported in full in Supplementary Table S1, which specifies the entire nested-tuning procedure. Across the seven learners the search comprised 21 candidate configurations; because every configuration was evaluated in all three inner folds of all 15 outer folds, the tuning procedure required 21 × 3 × 15 = 945 inner-loop pipeline fits, followed by 7 × 15 = 105 outer-fold refits of the selected configuration. The search space was specified as an explicit list of candidate configurations rather than as a full factorial product, and no random, Bayesian or adaptive search was used, so the procedure is exhaustive over the stated candidates and deterministic under the fixed seed.

### 2.5. Nested Cross-Validation

Generalisation performance was estimated with repeated nested cross-validation. The outer loop consisted of five folds stratified by event status and repeated three times, and it was used only for model evaluation. The inner loop consisted of three folds and was used only for hyperparameter selection. Because tuning and evaluation on the same data can lead to overly optimistic performance estimates, model selection was nested within the evaluation loop [24]. Instead of averaging patient-level predictions across repeats, we retained the full distribution of fold-level performance estimates and summarised them with a mean and standard deviation. This approach makes the variability of model performance more visible than a single train-test split. A fixed random seed (42) was used for all stochastic steps to ensure reproducibility.

### 2.6. Evaluation Metrics

Discrimination was assessed with Harrell’s concordance index [25] and Uno’s inverse-probability-of-censoring-weighted concordance, with the censoring distribution estimated from the training data only [26]. Time-dependent area under the curve (AUC) was calculated over a 12- to 60-month horizon. Overall prediction error was summarised with the integrated Brier score, using a time grid restricted to the overlap between training and test follow-up times [27]. Lower integrated Brier scores indicate better probabilistic accuracy. Because the Brier score attainable by an uninformative prediction in censored survival data is a function of the marginal event distribution and of the prediction horizon rather than a fixed constant, no universal noninformative benchmark was adopted. Time-dependent and integrated Brier scores are therefore reported descriptively. As an appropriate comparator we therefore fitted an explicit null model that assigns every patient the marginal Kaplan–Meier survival probability, evaluated on the identical time grid and with the identical censoring weights, and we report the time-dependent Brier scores against this cohort-specific and horizon-specific reference rather than against a fixed constant. In the absence of censoring the Brier score of such a null model at time t reduces to S(t) [1 − S(t)], which attains the value 0.25 only in the special case S(t) = 0.5 and is smaller at every other horizon; the null reference is consequently a curve rather than a line. The proportional reduction in Brier score relative to this null, that is 1 − (Brier of the model/Brier of the null), is reported as an index of prediction accuracy.

### 2.7. Calibration, Clinical Utility and Competing Risks

To keep calibration and decision analyses free of leakage, both were based on out-of-fold predictions so that each patient was scored only by models that had not been trained on that patient. Calibration at 36 months was assessed by grouping predicted survival probabilities into deciles and comparing them with Kaplan–Meier estimates of observed survival within each decile [28]. Clinical usefulness was examined with decision-curve analysis, which estimates the net benefit of model-based decisions relative to treat-all and treat-none strategies across a range of threshold probabilities [29]. Because death without recurrence can act as a competing event, Aalen–Johansen cumulative-incidence curves were also plotted as a descriptive sensitivity analysis. Interpretation was explicitly limited to the way the supplied RFS indicator encoded death. The Elastic-Net Cox model was used for the originally reported downstream calibration, decision-curve, risk-score, and permutation-importance displays.

### 2.8. Variable Importance Approach

To explore which variables contributed most to the recurrence signal, permutation importance was calculated on a model re-fitted to the full dataset [30]. This step was used for interpretation only and not for estimating model performance. The unbiased estimate of generalisation was taken only from the nested cross-validation analysis [20]. Because standard permutation importance is sensitive to correlated predictors, it is interpreted descriptively rather than as a conditional effect. To obtain a survival-specific and individualised attribution, SurvSHAP(t) was additionally computed [31]. SurvSHAP(t) decomposes the predicted survival function of each patient into time-dependent contributions of the individual predictors, so that an attribution is available at every follow-up time rather than as a single scalar. It was applied to the Elastic-Net Cox model at the same point in the workflow as permutation importance, that is, on a model re-fitted to the full dataset and for interpretation only, so the nested cross-validation estimates are unaffected. Because the sampling estimator is applied to the input space of the estimator, the preprocessing steps were fitted and applied once before the attribution step. The estimator was fitted to the full cohort of 356 patients, and all 356 patients were then explained with 4 Monte-Carlo sampling repetitions under the fixed seed (42); the attributions are pooled at patient level, that is, the mean absolute attribution across patients is taken at each follow-up time before integration. The number of sampling repetitions was limited by the computational cost of the sampling estimator, so the ranking separates the leading variables but is not intended to separate variables whose attributions are closely spaced.

### 2.9. Ablation and Sensitivity Analysis Approaches

Two robustness analyses were performed. First, an ablation analysis compared the full pipeline with a simpler variant in which the robust multivariable outlier-handling step was replaced by standard imputation and scaling, allowing the contribution of that component to be examined directly. Second, the outlier-handling configuration was varied by changing the chi-squared significance level and the robust z-score threshold, and the outer-loop concordance was recalculated for each combination. Small changes across these settings would suggest that the main findings were not driven by a particular treatment of extreme AFP values or by a narrow preprocessing choice.

### 2.10. Study Protocol, Ethical Considerations, and Funding

This study was conducted in accordance with the ethical principles of the Declaration of Helsinki and its subsequent amendments, and complied with all applicable institutional and national regulations governing research involving human participants. Ethical approval was obtained from the Inonu University Institutional Review Board for Non-Interventional Clinical Research (approval date: 30 June 2026; approval number: 10573). Given that this study was a secondary analysis of previously published anonymised data, no new participant contact or identifiable information was involved, and the requirement for individual informed consent was waived. This study was financially supported by the Inonu University Scientific Research Projects Coordination Unit (Grant ID: TSA-2026-5141). Reporting followed the TRIPOD + AI statement (Transparent Reporting of a multivariable prediction model for Individual Prognosis Or Diagnosis), an updated reporting guideline for clinical prediction models using regression or machine-learning methods [32].

### 2.11. Biostatistical Analysis

Continuous variables were summarised as median (interquartile range), and categorical variables were summarised as number (percentage). Exploratory group comparisons used the Mann–Whitney U test for continuous variables and the chi-squared test for categorical variables. The primary endpoint was analysed as time-to-event recurrence-free survival using the observed event indicator and event time. Model development and evaluation followed repeated nested cross-validation, with all data-dependent preprocessing and hyperparameter selection restricted to training folds. Discrimination and prediction error were assessed with Harrell’s concordance index, Uno’s inverse-probability-of-censoring-weighted concordance, time-dependent AUC, and the integrated Brier score. Calibration at 36 months, decision-curve analysis, and Aalen–Johansen cumulative-incidence analysis were based on out-of-fold predictions or fold-specific test data, as appropriate. Permutation importance, ablation analysis, and threshold-sensitivity analysis were treated as secondary explanatory or robustness analyses rather than as independent estimates of model generalisation.

### 2.12. Software and Reproducibility

All analyses were performed in Python 3.12 using scikit-survival 0.27.0 [21], scikit-learn 1.8.0, NumPy 2.0.2, SciPy 1.16.3, and pandas 2.2.2. The SurvSHAP(t) analysis used the survshap package (version 0.4.2). All random processes were run with a fixed seed (42), and the full pipeline, including the fold definitions, was reproducible from the analysis notebook. The analysis workflow was fully scripted, and the same notebook was used to generate the reported results.

### 2.13. Additional Validation and Sensitivity Analysis Approaches

Additional analyses used the same cohort, outcome definitions, and random seed (42). Model comparisons were based on three repeats of five outer folds with three inner folds. The full predictor set was contrasted with a reduced-availability proxy set to examine how restricting variables that could plausibly be available before transplantation affected discrimination. Because the timing of predictor collection was not documented in the supplied dataset, this reduced set was treated as a sensitivity analysis rather than a validated pre-transplant model. Dependence among estimates from repeated cross-validation was addressed with the Nadeau–Bengio corrected repeated k-fold t test, using the correction factor 1/15 + 1/4 and 14 degrees of freedom. Calibration was evaluated at 12, 24, 36, 48, and 60 months using first-repeat out-of-fold predictions grouped into quintiles. A single, prespecified repeat was used for this analysis because each repeat of the outer loop yields exactly one complete set of out-of-fold predictions in which every patient is scored once by a model that was not trained on that patient. Pooling the three repeats would enter each patient three times into the same grouped Kaplan–Meier calibration estimator and would understate the width of the observed-risk estimates, whereas averaging the predictions across repeats would produce a smoothed three-model ensemble prediction that none of the benchmarked learners actually generates. Restricting the calibration analysis to the first repeat therefore preserves the one-prediction-per-patient structure that the estimator assumes and avoids implicit ensembling; the remaining two repeats are retained in the discrimination analysis, where they contribute to the reported fold-level dispersion. Proportional-hazards assumptions were assessed with rank-transformed Schoenfeld residual tests. To examine the practical effect of one common leakage scenario, a sensitivity analysis applied global preprocessing and non-nested tuning; this comparison was interpreted descriptively. An additional full-data outlier audit used Minimum Covariance Determinant distance at the 0.975 chi-squared cutoff and cell-wise |robust z| > 3. This audit was used only to describe the prevalence and distribution of extreme values and not to estimate model generalisation.

## 3. Results

### 3.1. Cohort Characteristics

The cohort comprised 356 patients, of whom 183 (51.4%) experienced recurrence. Median observed follow-up time was 52 months (reverse Kaplan–Meier median follow-up 65 months on the recurrence-free survival scale). As shown in Table 1, the population was predominantly male and hepatitis B-positive, and cirrhosis was present in 87.6% of patients. Patients who developed recurrence differed markedly from those who did not across the expected markers of tumour burden and tumour aggressiveness. In addition to the major tumour-related variables, patients with recurrence were slightly younger and had higher CA 19-9 levels, greater intraoperative blood loss, and higher transfusion requirements. Median α-fetoprotein was substantially higher in the recurrence group than in the non-recurrence group (193.5 vs. 17.5 ng/mL), and maximum tumour diameter was more than twofold greater (6.5 vs. 3.0 cm). Microvascular invasion, portal-vein tumour thrombus, satellite lesions, and poor differentiation were also much more frequent among patients with recurrence (all *p* < 0.001). In contrast, pre-transplant interventions were not significantly associated with recurrence. Overall, descriptive differences were more pronounced for tumour-biology variables than for pre-transplant treatment history.

### 3.2. Discrimination and Calibration

**Figure 1:**
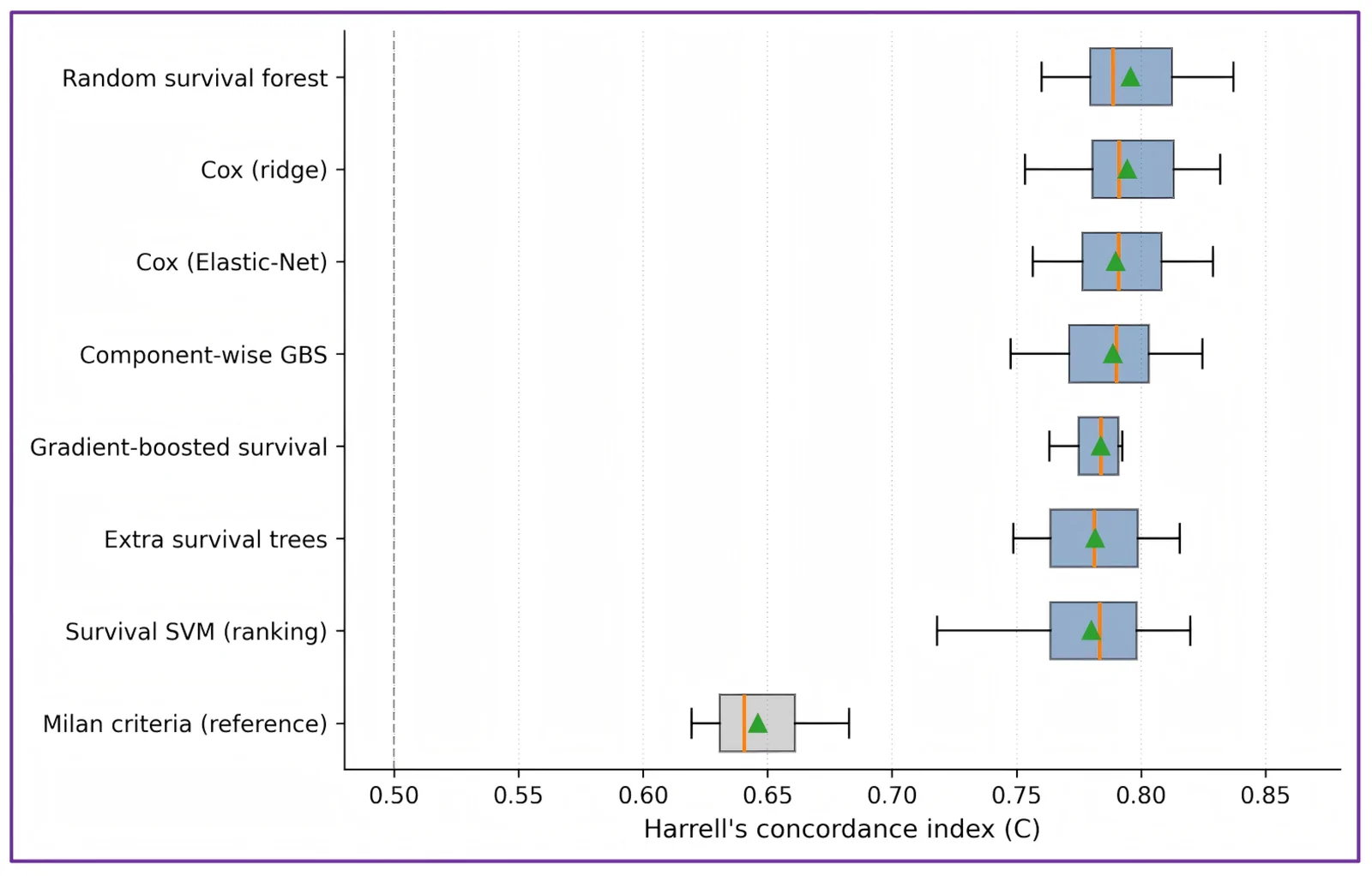
Distribution of Harrell’s concordance index across outer cross-validation folds for each learner. Boxes show the interquartile range, the orange line the median and the green triangle the mean; the dashed line marks chance-level discrimination (0.5). All survival learners cluster tightly above 0.78, whereas the Milan criteria fall to approximately 0.68.

Under the leakage-free design, discrimination was consistent across the seven survival learners, with Harrell concordance values ranging from 0.78 to 0.80 (Table 2; Figure 1). Random survival forest showed the highest discrimination (Harrell C 0.80 ± 0.02; Uno C 0.80 ± 0.02), followed closely by the ridge-penalised Cox model (Harrell C 0.80 ± 0.02) and the Elastic-Net Cox model (Harrell C 0.79 ± 0.02). The overlap in performance suggests that no single modelling strategy was clearly dominant and that the more flexible tree-based methods offered little practical advantage over regularised Cox models. The remaining learners clustered within the same narrow range, so discrimination was stable but moderate across all model classes.

**Figure 2:**
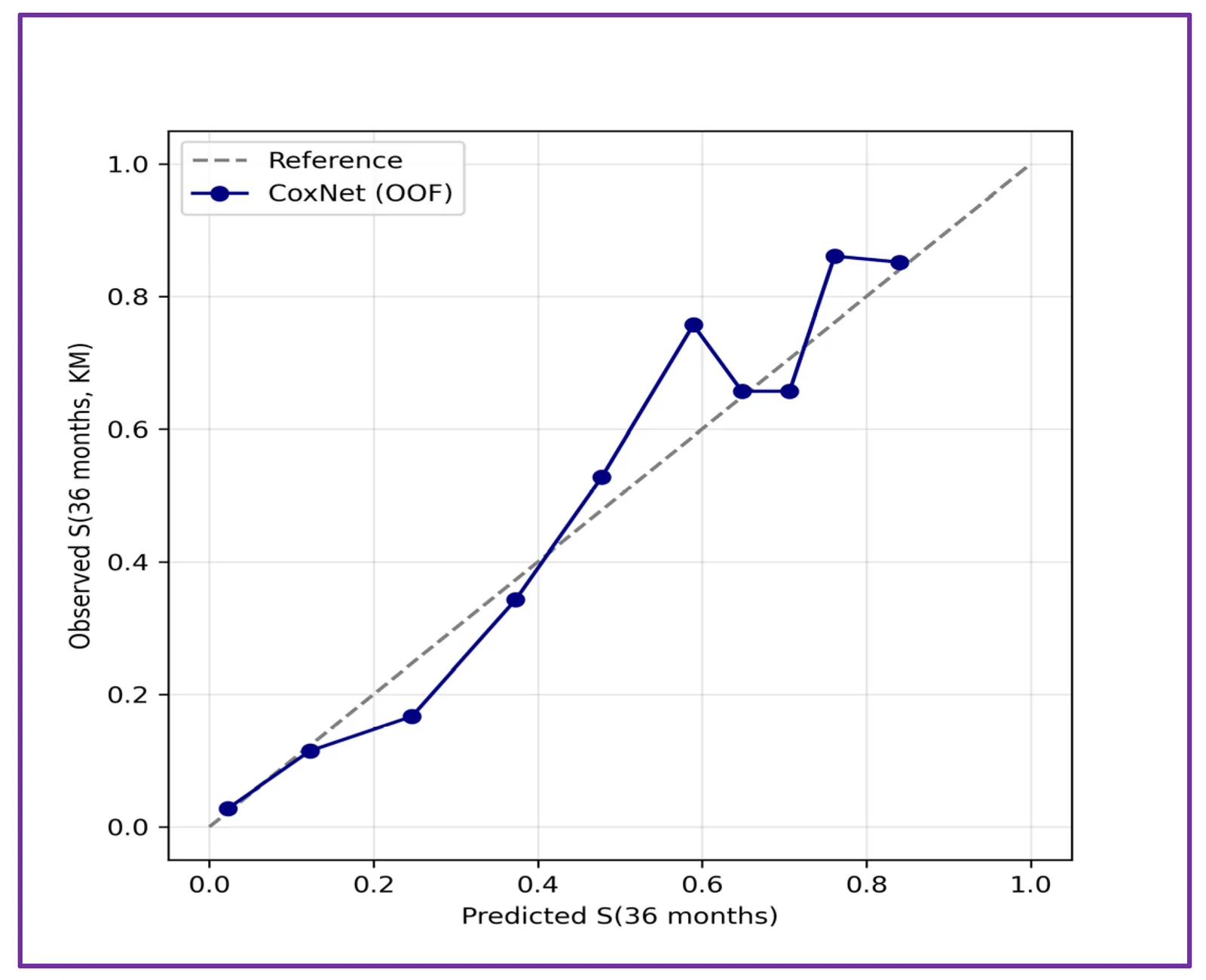
Calibration of predicted 36-month survival probabilities against Kaplan–Meier estimates of observed survival, based on out-of-fold predictions for the Elastic-Net Cox (CoxNet) model. Proximity to the line of identity indicates acceptable calibration.

All learners outperformed the Milan criteria, which achieved a Harrell C of 0.68 ± 0.03 on the same outer folds. The absolute gain of about 0.12 in concordance suggests improved discrimination relative to the fixed morphological rule, while the overall level of discrimination also shows that post-transplant recurrence cannot be predicted with near-perfect accuracy. At 60 months, random survival forest reached a time-dependent AUC of 0.86 ± 0.04 and an integrated Brier score of 0.16 ± 0.01. The Elastic-Net Cox model showed similar overall performance, with Harrell C 0.79 ± 0.02 and an integrated Brier score of 0.16 ± 0.01. Calibration of the Elastic-Net Cox (CoxNet) model at 36 months, based on out-of-fold predictions, was close to the line of identity, with only mild deviation in the middle deciles (Figure 2). This pattern supports acceptable agreement between predicted and observed risk in out-of-sample data.

### 3.3. Clinical Utility and Risk Separation

**Figure 3:**
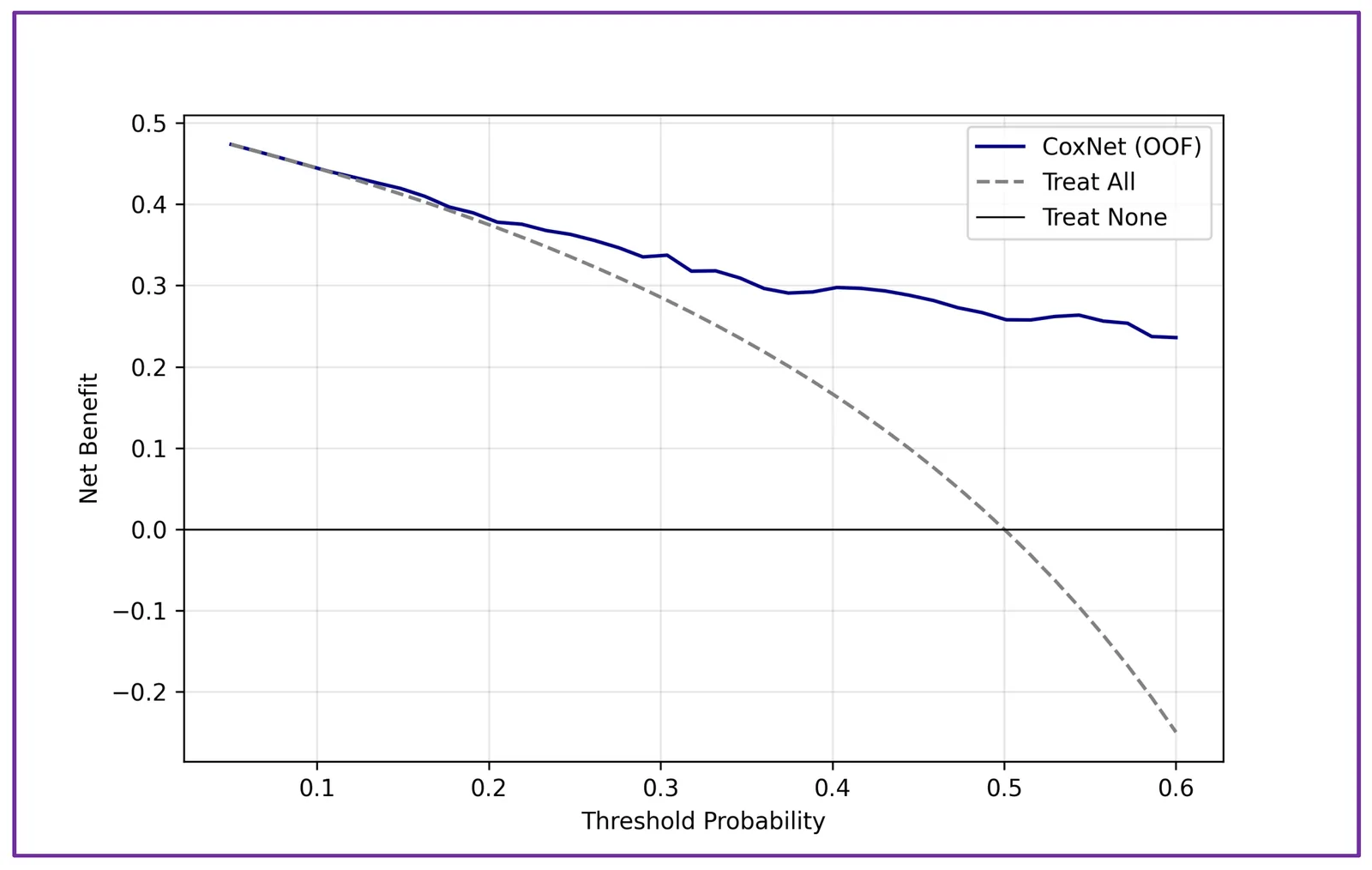
Decision-curve analysis at 36 months. Net benefit of the Elastic-Net Cox (CoxNet) model (solid line) is compared with the treat-all (dashed) and treat-none (horizontal) strategies across threshold probabilities. The model provides positive net benefit throughout the clinically relevant range.

**Figure 4:**
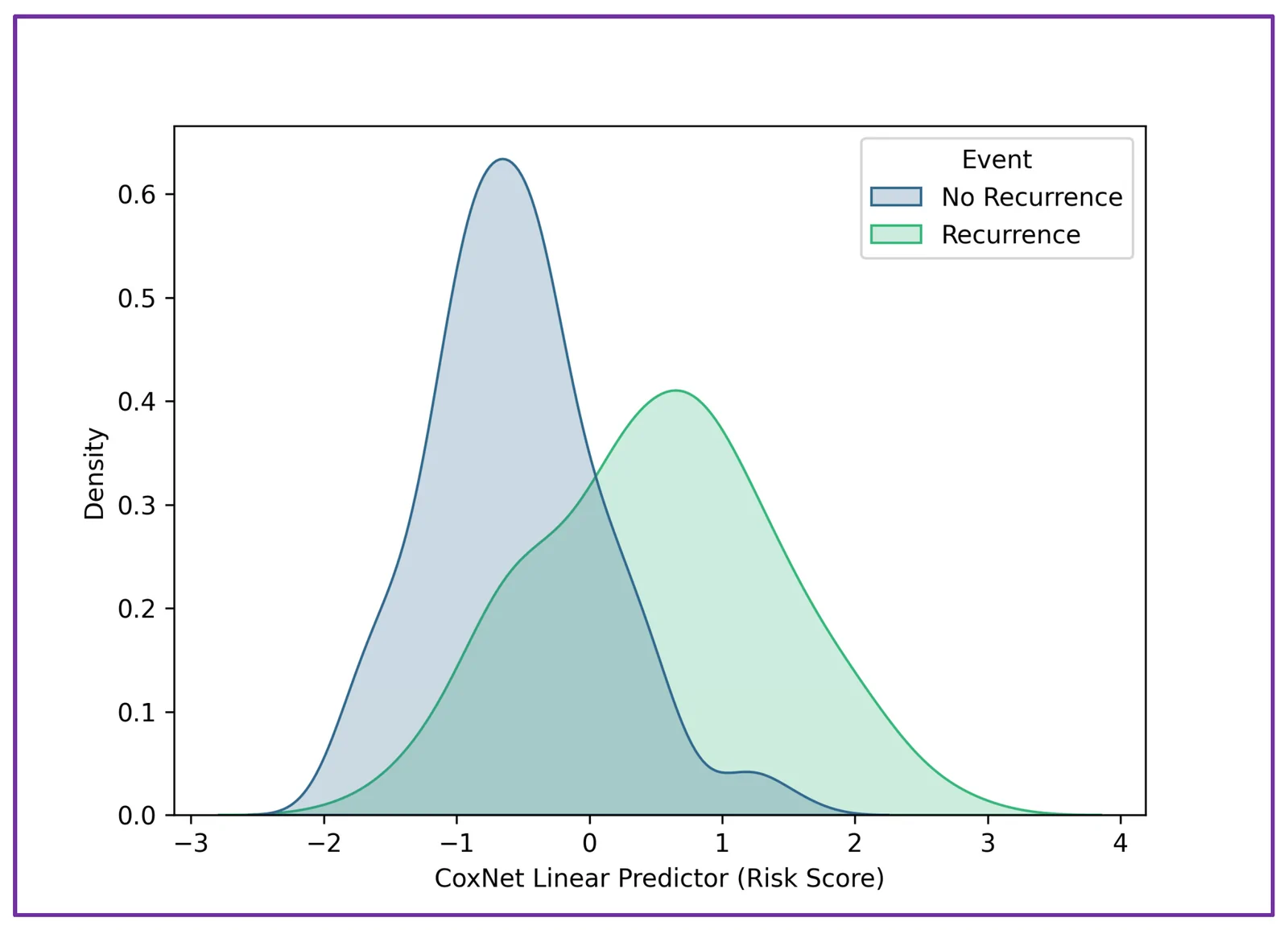
Distribution of the model’s linear predictor (risk score) for patients with and without recurrence. The upward shift for recurrent cases, with partial overlap, mirrors the moderate discrimination quantified by the concordance index.

**Figure 5:**
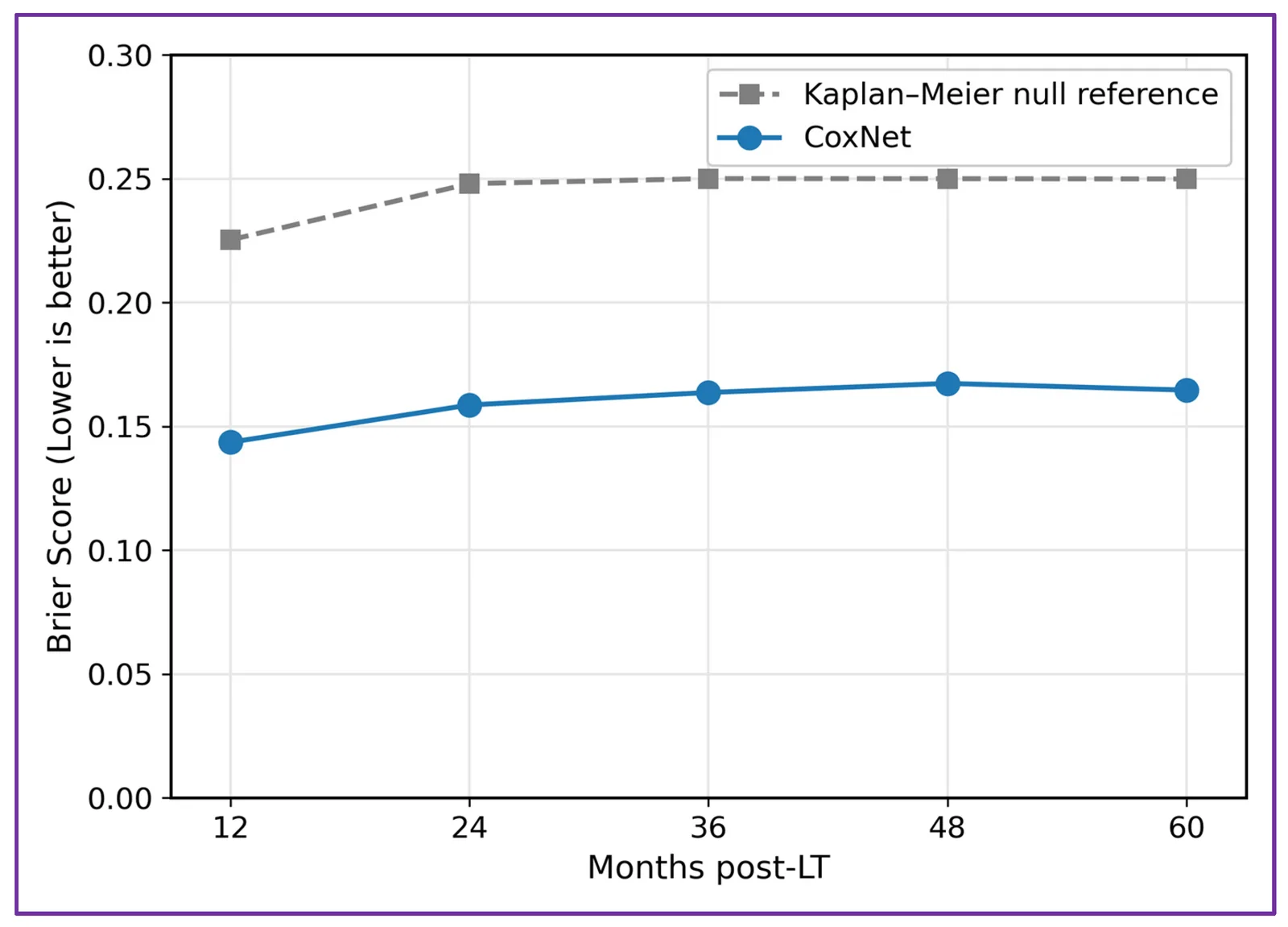
Time-dependent prediction error (Brier score) from 12 to 60 months after transplantation. The dashed grey curve is the Kaplan–Meier null reference, that is, the Brier score of a null model assigning every patient the marginal survival probability, evaluated on the same time grid and with the same censoring weights. This reference is time-dependent rather than constant, rising from 0.225 at 12 months to 0.250 from 36 months onwards as the marginal survival probability approaches 0.5, and it replaces the fixed value of 0.25 shown previously, which is valid only where survival equals 0.5. The separation between the two curves corresponds to proportional reductions in Brier score of 33.1 to 36.3 per cent across the window.

Decision-curve analysis showed that the Elastic-Net Cox (CoxNet) model provided positive net benefit across clinically relevant threshold probabilities and performed better than both the treat-all and treat-none strategies (Figure 3). The separation between the model curve and the treat-all strategy became more pronounced at higher thresholds, suggesting greater usefulness when unnecessary intervention carries a meaningful cost. This clinical utility was consistent with the distribution of model-derived risk scores (Figure 4). Patients who developed recurrence had higher linear predictor values than those who remained recurrence-free, although the two groups still showed substantial overlap. This degree of separation is consistent with the moderate but reproducible discrimination observed in cross-validation. In addition, the time-dependent Brier score remained between 0.144 and 0.167 from 12 to 60 months, so that probabilistic accuracy was stable throughout the follow-up window (Figure 5). Because the Brier score attainable by an uninformative model in censored data changes with the event distribution and with the prediction horizon, these values are interpreted against an explicit Kaplan–Meier null reference rather than against a fixed constant. In this cohort that null reference is itself time-dependent, rising from 0.225 at 12 months to 0.248 at 24 months and to 0.250 from 36 months onwards as the marginal survival probability approaches 0.5. The corresponding proportional reductions in Brier score achieved by the Elastic-Net Cox model were 36.3, 36.0, 34.5, 33.1 and 34.1 per cent at 12, 24, 36, 48 and 60 months. The model therefore improves on an uninformative prediction by approximately one third at every horizon, and it does so even at 12 months, where the null itself is most accurate because few patients have recurred; that stability is visible only against a horizon-specific reference and is concealed by a fixed benchmark. The same narrow Brier range was obtained for every learner yielding a survival function (Table 2).

### 3.4. Competing-Risk Incidence

**Figure 6:**
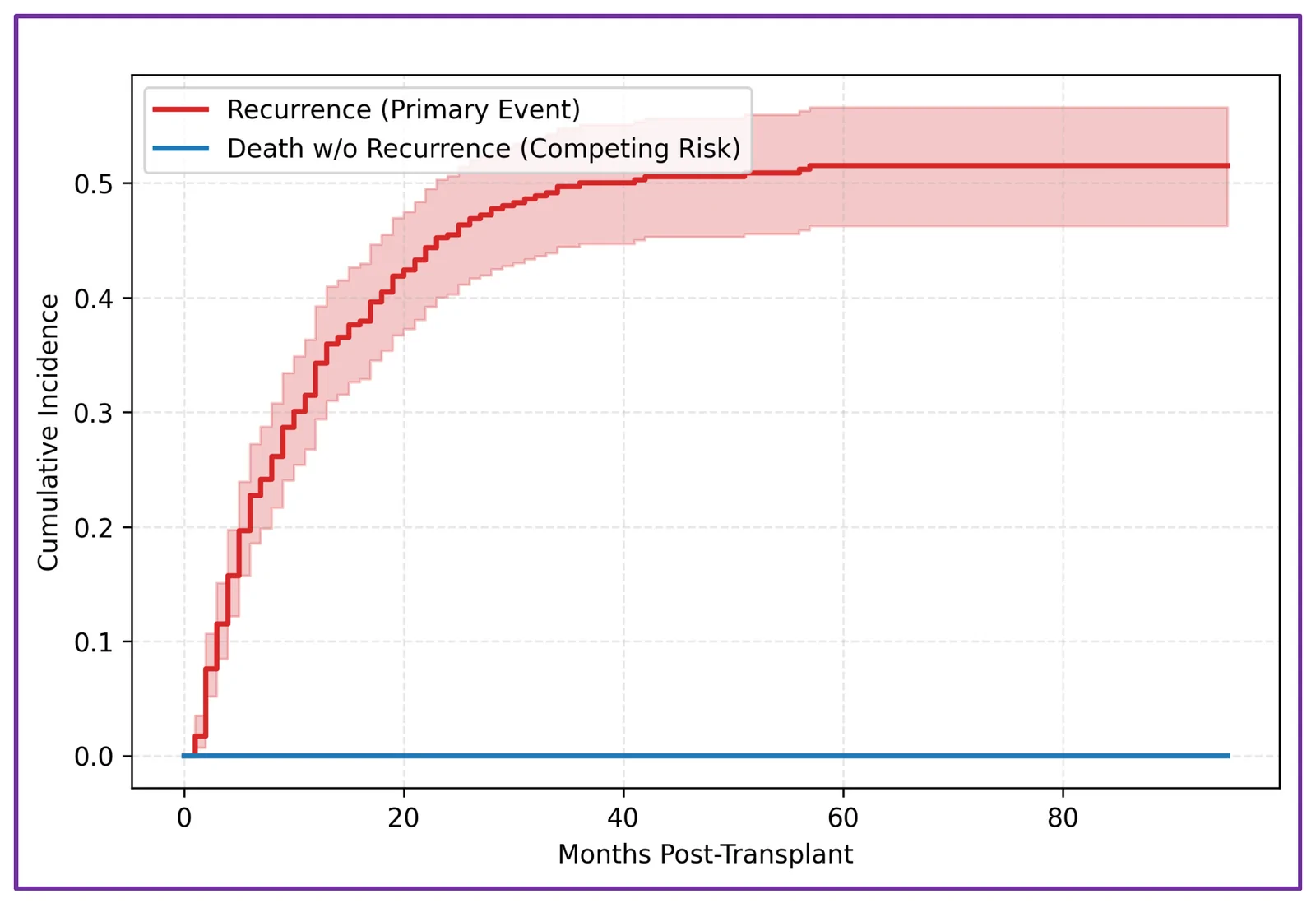
Aalen–Johansen cumulative incidence of recurrence (primary event) and death without recurrence (competing event), with pointwise confidence bands. No death without recurrence was observed, so the cumulative-incidence estimate for recurrence reduces to the complement of the Kaplan–Meier estimate and the curve is shown for completeness rather than as an independent competing-risk result. The shaded red area denotes the pointwise confidence interval for the recurrence cumulative incidence.

Aalen–Johansen cumulative-incidence analysis showed that recurrence increased steeply during the first two years after transplantation and then approached a plateau of approximately 0.51 (Figure 6). In contrast, the cumulative incidence of death without recurrence remained close to zero. In this dataset, the supplied recurrence-free survival indicator therefore appears to behave as though death was absorbed into the main event rather than coded as a separate competing outcome. This finding is reported descriptively because its interpretation depends on how the original endpoint was defined.

### 3.5. Variable Importance

**Figure 7:**
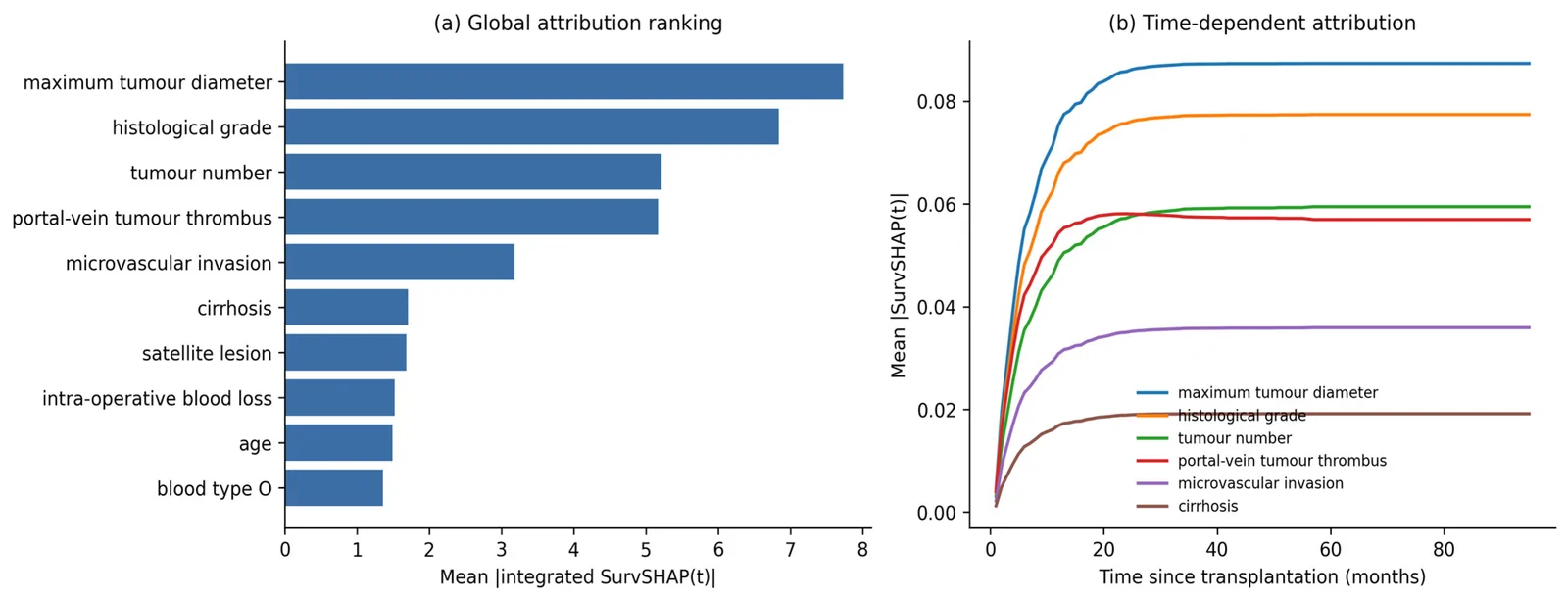
SurvSHAP(t) attributions for the Elastic-Net Cox model. The model was re-fitted to the full dataset of 356 patients, and all 356 patients were explained with 4 sampling repetitions under the fixed seed, with the attributions pooled at patient level. (**a**) Global ranking by mean absolute integrated attribution, ten largest values. (**b**) Mean absolute attribution as a function of time since transplantation for the six leading predictors. Attribution magnitude increases over the first two years and then plateaus for every predictor, so the rise is a property of the separating survival curves rather than a variable-specific time-varying effect.

Permutation importance, calculated for interpretation on a model re-fitted to the full dataset, identified maximum tumour diameter as the most influential predictor, followed by α-fetoprotein. Tumour number, the location of portal-vein tumour thrombus, histological grade, and CA 19-9 contributed at a lower level, whereas operative and demographic variables had relatively little influence. This ranking was consistent with the descriptive comparisons in Table 1, in which the same tumour-related variables showed the strongest differences between the recurrence and non-recurrence groups. SurvSHAP(t) was computed for all 356 patients with four sampling repetitions and produced a broadly concordant ranking. The largest mean absolute integrated attributions were maximum tumour diameter (7.73), histological grade (6.84), tumour number (5.21), portal-vein tumour thrombus (5.17), and microvascular invasion (3.18); cirrhosis (1.71), satellite lesions (1.68), and intraoperative blood loss (1.52) followed at a much lower level (Figure 7a). The two rankings agree on maximum tumour diameter as the leading predictor and on the dominance of tumour-burden variables, but they differ in the position of α-fetoprotein, which is second under permutation importance and twelfth under SurvSHAP(t). The two measures answer different questions, permutation importance quantifying the loss in model performance when a variable is disturbed and SurvSHAP(t) quantifying the average size of a variable’s contribution to individual predicted survival functions, so agreement on the leading variables and disagreement on a strongly skewed marker such as α-fetoprotein is not contradictory. The time-dependent profiles (Figure 7b) show that attribution magnitudes rise over the first two years and then plateau, and that this rise is common to all predictors: the ratio of late (≥48 months) to early (≤24 months) mean absolute attribution lies between 1.20 and 1.37 for the five leading variables and between 1.18 and 1.37 across the ten largest. Histological grade lies inside that range (1.33) and is not the highest, since tumour number reaches 1.37, and when the profiles are normalised to the total attribution at each time point the relative share of histological grade rises only from 15.1 to 15.7 per cent, a change of 0.6 percentage points. SurvSHAP(t) therefore provides at most weak support for the variable-specific departure from proportionality suggested by the Schoenfeld test, and the increase in attribution magnitude is better read as a property of the separating survival curves than as evidence of a grade-specific time-varying effect. Because the attribution step used four sampling repetitions, this analysis is descriptive, and the ordering of closely spaced variables should not be over-interpreted.

### 3.6. Ablation and Sensitivity Analysis Results

The ablation analysis showed that the robust multivariable outlier-handling step had little effect on discrimination. The full pipeline achieved a Harrell C of 0.793 ± 0.023, compared with 0.795 ± 0.023 when this component was replaced by standard imputation and scaling (Table 3). The threshold-sensitivity analysis also showed minimal variation, with concordance values between 0.793 and 0.794 across the tested combinations of chi-squared significance level and robust z-score threshold. Together, these findings suggest that model performance was stable and was not materially driven by the handling of extreme values, including the anomalous α-fetoprotein observations identified during data checking. The selected hyperparameters were also broadly consistent with moderate model complexity across learners, as summarised in Table 4. The separate full-data audit flagged 150 records and 159 cells: AFP 73, CA 19-9 37, maximum tumour diameter 28, and blood loss 21. The largest AFP was 275,924 ng/mL (ID 323); two values exceeded 60,500 ng/mL and 15 values were exactly 60,500 ng/mL. Fold-specific counts may differ because preprocessing is re-fitted within training folds.

### 3.7. Additional Validation and Sensitivity Analysis Results

In the additional analysis, mean Harrell C for the full predictor set was 0.796 for ridge Cox and 0.796 for random survival forest, with fold-level dispersion as reported in Table 2, and 0.677 ± 0.029 for the Milan criteria. The differences below are paired fold-level contrasts and are therefore not obtained by subtracting the rounded means. Under the corrected repeated k-fold test, the random survival forest-minus-Cox difference was −0.0005 (95% CI −0.0184 to 0.0175; *p* = 0.957), providing no evidence of a difference. The Cox-minus-Milan difference was 0.119 (95% CI 0.076 to 0.161; *p* < 0.001).

For the reduced-availability proxy set, mean Harrell C was 0.746 ± 0.026 for Cox and 0.770 ± 0.029 for random survival forest. Relative to the full predictor set, concordance decreased by 0.050 for Cox and 0.026 for random survival forest. Under the corrected repeated k-fold test, the random survival forest-minus-Cox difference was 0.023 (95% CI −0.003 to 0.049; *p* = 0.073). Because predictor measurement times were not documented, these results represent a variable-restriction sensitivity analysis rather than validation of a pre-transplant model.

Five-group mean absolute calibration error was 0.055, 0.057, 0.059, 0.055, and 0.057 at 12, 24, 36, 48, and 60 months; maximum group deviations were 0.096, 0.139, 0.131, 0.135, and 0.144. Among the variables tested, histological grade showed the strongest departure from proportionality (rank-transformed Schoenfeld *p* = 0.0149), which is consistent with a possible time-varying effect.

The tested leakage-prone scenario produced a negligible mean change in concordance (leaky-minus-nested, −0.0003). This was retained as a descriptive sensitivity analysis rather than a formal inferential comparison. Because the analysed numerical predictors had no missing values and global standardisation has limited effect on rank-based Cox discrimination, this scenario was not expected to create strong inflation; the result does not imply that leakage is generally harmless. An ID-ordered 70/30 stress test yielded C = 0.778; because ID is not a verified date or era variable, this was not interpreted as temporal validation.

## 4. Discussion

Survival learners showed moderate but consistent discrimination for post-transplant recurrence, with Harrell C values clustered between 0.78 and 0.80. Random survival forest yielded the highest mean concordance in the primary benchmark, but its performance was closely matched by the ridge-penalised Cox model and the Elastic-Net Cox model. In the dependency-corrected repeated k-fold comparison, full-feature Cox had higher concordance than Milan, whereas RSF and Cox were statistically indistinguishable. Calibration at 36 months was acceptable, and decision-curve analysis showed positive net benefit across clinically relevant thresholds. The leading predictors were biologically plausible, particularly maximum tumour diameter and alpha-fetoprotein. Taken together, the findings support two conclusions. First, recurrence-risk modelling adds information beyond a fixed morphological rule within this cohort. Second, under a strict leakage-free framework, attainable performance appears moderate rather than near-perfect. Read against the three hypotheses stated in the Introduction, the first was confirmed: attainable concordance was moderate, close to 0.80, and clearly below the accuracy of 0.95 reported for the derived-risk-label classification of the same cohort, although the two figures describe different prediction tasks and are not directly comparable. The second was also confirmed: the seven learners spanned a range of about 0.02 in concordance, and the dependency-corrected comparison of the random survival forest with the Cox model gave no evidence of a difference. The third was supported for the contrast that was formally tested, in which the full-feature Cox model had higher concordance than the Milan criteria, and descriptively for the remaining learners; it was therefore not refuted, although it was not tested learner by learner.

The most informative aspect of the present study is the contrast with the previously published deep-learning re-analysis of the same cohort [13]. In that report, patients were first assigned to high- and low-risk groups on the basis of tumour size, grade, and number, and a classifier was then trained to reproduce those derived labels. That analysis reported a classification accuracy of 0.95, compared with 0.86 for the Milan criteria, but those values were obtained for a derived risk-label task rather than direct event-time prediction [13]. By contrast, the present analysis modelled the observed recurrence event and follow-up time directly. This distinction is methodologically important. As discussed in the leakage literature, prediction targets that are constructed from the input features can produce overly optimistic estimates because the model is effectively rewarded for recovering a rule that was already embedded in the data structure [20]. In addition, preprocessing steps that depend on the full dataset and the failure to separate model tuning from final evaluation can further inflate apparent performance [20,24]. For that reason, the lower but more stable estimates observed here are more relevant than a headline accuracy obtained from a simplified target. In practical terms, the present study suggests that, in tabular transplant-oncology data of this size, methodological rigour has a greater effect on credibility than increasing architectural complexity.

The performance observed here is also in line with the broader literature when models are evaluated under more realistic conditions. In a multicentre cohort study, the pre-DeepSurv model showed external-validation concordance values in the 0.77–0.82 range, whereas the post-DeepSurv model, after clinicopathologic variables were added, improved further to approximately 0.83–0.84 in external validation [8]. In a single-centre living-donor cohort, a pre-transplant AI model achieved an AUC in the validation cohort of 0.717, outperforming the Milan criteria (AUC 0.646). When histological grade was added, the AUC in the validation cohort increased to 0.771, again illustrating the difference between a strictly preoperative problem and a post-pathology or post-transplant one [7]. Registry-based and externally validated machine-learning studies have shown a similar pattern, with moderate rather than near-perfect discrimination in more realistic validation settings [9,19]. By contrast, imaging-driven radiomics and deep-learning models have sometimes reported higher values, particularly for early recurrence, but these studies often target a narrower endpoint, use different feature spaces, or depend on imaging workflows whose generalisability across scanners and centres remains uncertain [12,15,16,17]. This broader context is important for interpreting the results of the present study. The best model achieved a concordance of about 0.80. Although this value may seem lower than the more optimistic results reported in studies based on single-split classification or derived risk labels, those approaches address a different prediction task. By directly modelling the observed recurrence event and follow-up time within a leakage-free survival framework, the present analysis provides a more clinically relevant estimate of performance. The present result is therefore consistent with the heterogeneity of post-transplant HCC recurrence and with the range reported in independent studies using structured clinical data, suggesting that it may be close to a realistic ceiling for clinically usable prediction in this setting.

From a clinical perspective, the present findings are relevant mainly to recurrence-risk stratification rather than to pure pre-transplant candidate selection. This distinction should be kept explicit, particularly in a field that has progressively moved from fixed morphological rules toward combined morphological and biological models of recurrence [2,3]. The predictor set in the present analysis included not only baseline and treatment variables but also pathological and perioperative information, such as microvascular invasion, histological grade, portal-vein tumour thrombus, and operative blood loss. Models built with these variables are therefore better suited to post-transplant risk stratification and surveillance planning than to decisions made before transplantation. Even within that context, however, the results remain clinically meaningful. The Milan criteria summarise tumour burden using a binary morphological threshold, whereas the current models provide a continuous estimate of risk and showed better discrimination, reasonable calibration, and positive net benefit. The fact that tumour size and α-fetoprotein emerged as the dominant contributors is also clinically intuitive and consistent with the prior transplant-oncology literature, in which these variables repeatedly remain central even when more complex model structures are used [7,9,12]. These variables are routinely available, and their strong contribution supports the biological face validity of the models, even though the full prediction framework should be interpreted as explant-informed rather than purely preoperative. The lower discrimination of the pre-transplant proxy analysis empirically reinforces that the Milan comparison is contextual and not replacement evidence for candidate selection.

The main strengths of this study are methodological. All data-dependent preprocessing steps were confined to the training folds, hyperparameter tuning was separated from final evaluation through nested cross-validation, and performance was described using a broader set of measures than discrimination alone. Reporting calibration, decision-curve analysis, competing-risk incidence, and prediction error alongside concordance gives a more clinically useful picture of model behaviour than Harrell C alone. In addition, the ablation and threshold-sensitivity analyses showed that the main findings were not materially driven by the handling of extreme values. This is particularly relevant because the public dataset contained an anomalously high α-fetoprotein value compared with the maximum reported in the source publication. The consistency of results across these analyses increases confidence that the main conclusions are robust.

### Limitations

The principal limitation is the absence of external validation. No independent cohort was available to us, so every performance estimate reported here is internal to a single tertiary centre and to one previously published dataset, and none of them establishes transportability. Repeated nested cross-validation protects against optimism that arises from tuning and preprocessing on the evaluation data, but it cannot detect the shift in case mix, surgical practice, allocation policy or aetiological background that separates one centre from another, and it is that shift, rather than internal optimism, that most often erodes the performance of transplant-oncology models in new settings. The ID-ordered analysis reported in Section 3.7 is a sensitivity check on data ordering and is not a substitute for external validation, because the supplied file carries no admission or surgery dates and the ordering therefore has no verified temporal meaning. The concordance values reported here should accordingly be read as an internally valid characterisation of what these learners achieve on this cohort under a leakage-free design, and not as an estimate of what they would achieve elsewhere. Confirmation in independent multicentre data is required before any of the compared models, including the Milan reference, is used to inform surveillance intensity for an individual patient. The proportional-hazards diagnostics identified a possible non-proportional effect for histological grade. The corrected repeated k-fold test was used because outer-fold estimates from repeated cross-validation are dependent; nevertheless, inference remains conditional on this resampling design and cohort. With 183 recurrence events and 25 candidate predictors, the ratio of events to candidate predictors is about 7, below the conventional value of 10; penalised estimation and nested cross-validation limit but do not remove the resulting risk of optimism. The reduced-availability proxy set reflects plausible availability rather than verified collection timing. The ID-ordered split is an ordering sensitivity analysis and not temporal validation. The leakage analysis represents only one relatively weak leakage scenario and was interpreted descriptively. Prediction error is benchmarked against a cohort-specific Kaplan–Meier null model rather than a fixed constant, because the null value of the Brier score in censored survival data depends on the event distribution and on the prediction horizon. The resulting proportional reductions are specific to the event rate and follow-up window of this cohort and should not be compared directly with Brier scores, or with reductions in Brier score, reported for cohorts in which either differs. The full-data Minimum Covariance Determinant audit is not identical to fold-specific preprocessing and should not be interpreted as the number of replacements made in the primary model. SurvSHAP(t) was computed and is reported in Section 3.5, but conditional importance was not, so permutation importance should still be read as a descriptive ranking rather than as a conditional explanation. The SurvSHAP(t) attributions were computed with four Monte-Carlo sampling repetitions, a setting chosen for computational feasibility, so the leading variables are clearly separated but the ordering of closely spaced variables is not established by this run.

These findings have implications for future work. The immediate priority is prospective or external validation in independent cohorts, ideally across centres with different etiologic backgrounds and allocation practices. It would also be valuable to examine whether the addition of dynamic post-transplant information improves risk estimation beyond the ceiling observed here, as suggested by studies that distinguish between preoperative and postoperative model configurations [7,8]. At the same time, the imaging and radiomics literature suggests that future models may benefit from carefully integrated multimodal information if the gain in discrimination is weighed against complexity, reproducibility, and generalisability [15,17]. In transplant oncology, as in other clinical machine-learning settings, model development should begin with a clinically coherent outcome definition and a leakage-resistant evaluation design. Without that foundation, apparent performance may look impressive while remaining difficult to trust or deploy. If externally validated, an individualised post-transplant risk estimate could be presented through a monitored decision-support interface or risk calculator; prespecified thresholds, external calibration, workflow testing, and prospective impact evaluation would be required.

## 5. Conclusions

In this secondary analysis of a previously published single-centre LT-HCC cohort, leakage-free survival modelling achieved moderate but credible prediction of post-transplant recurrence, with a best-model concordance of 0.80, acceptable calibration, and positive clinical net benefit. All evaluated learners showed higher concordance than the Milan criteria in this cohort, but the difference between model classes was small, and simple penalised Cox models performed as well as more complex ensemble methods. These findings suggest that, in this setting, careful study design and leakage-resistant evaluation are more important than architectural complexity. They also indicate that recurrence-risk estimation can improve on fixed morphological criteria, particularly when used for post-transplant risk stratification rather than as a pure pre-transplant selection tool. More broadly, these findings support the use of leakage-free survival analysis as a practical methodological standard for future prognostic modelling studies in transplant oncology. All three prespecified hypotheses were therefore supported: discrimination was moderate rather than near-perfect, the learners were largely interchangeable, and every learner exceeded the Milan criteria in this cohort.

## Figures and Tables

**Table 1:** Baseline characteristics of the study cohort, overall and stratified by recurrence status. Continuous variables are summarised as median (interquartile range) and categorical variables as n (%). Descriptive *p*-values are from the Mann–Whitney U test (continuous) and the chi-squared test (categorical) and are provided for orientation only; the predictive analysis is based on the multivariable survival models. Sex coding follows the source dataset.

Characteristics	Overall (*n* = 356)	Recurrence (*n* = 183)	No Recurrence (*n* = 173)	*p*
Age (years)	52.0 (45.0–58.2)	51.0 (44.0–58.0)	54.0 (47.0–60.0)	0.014
AFP (ng/mL)	58.5 (6.8–1016.5)	193.5 (14.8–3921.5)	17.5 (5.0–114.5)	<0.001
CA 19-9, U/mL	25.1 (12.1–47.7)	28.5 (12.4–61.4)	21.9 (12.0–38.0)	0.026
Max tumour diameter (cm)	4.0 (2.5–8.0)	6.5 (3.5–11.0)	3.0 (2.0–4.5)	<0.001
Intraoperative blood loss (mL)	500 (300–800)	500 (400–800)	400 (300–600)	<0.001
Blood transfusion (mL)	0 (0–800)	600 (0–1000)	0 (0–800)	<0.001
Sex (male)	315 (88.5)	162 (88.5)	153 (88.4)	1.000
HBV	322 (90.4)	164 (89.6)	158 (91.3)	0.712
HCV	8 (2.2)	4 (2.2)	4 (2.3)	1.000
Cirrhosis	312 (87.6)	154 (84.2)	158 (91.3)	0.058
Multiple tumours	161 (45.2)	107 (58.5)	54 (31.2)	<0.001
Satellite lesion	54 (15.2)	45 (24.6)	9 (5.2)	<0.001
Microvascular invasion	151 (42.4)	106 (57.9)	45 (26.0)	<0.001
Portal-vein tumour thrombus	74 (20.8)	64 (35.0)	10 (5.8)	<0.001
Biliary tumour thrombus	21 (5.9)	17 (9.3)	4 (2.3)	0.010
Vena-cava invasion	12 (3.4)	12 (6.6)	0 (0.0)	0.002
Poor differentiation (≥II–III)	134 (37.6)	91 (49.7)	43 (24.9)	<0.001
Prior TACE	71 (19.9)	39 (21.3)	32 (18.5)	0.595
Prior radiofrequency ablation	30 (8.4)	16 (8.7)	14 (8.1)	0.976
Prior liver resection	51 (14.3)	29 (15.8)	22 (12.7)	0.489

TACE, transarterial chemoembolisation. Continuous variables are reported as median (interquartile range).

**Table 2:** Discrimination and overall accuracy by model, estimated from repeated nested cross-validation (three repeats of a five-fold outer loop). Values are mean ± standard deviation across outer folds. Harrell C, Harrell concordance index; Uno C, inverse-probability-of-censoring-weighted concordance; AUC, time-dependent area under the curve; IBS, integrated Brier score (lower is better). The ranking survival support vector machine does not yield a survival function, so its IBS is not defined; the Milan criteria provide concordance only. Values are rounded to two decimal places, consistent with the precision indicated by the fold-level standard deviations. The model comparisons in Section 3.7 are computed from unrounded fold-level estimates and are therefore not obtained by subtracting the rounded means.

Models	Harrell C	Uno C	AUC at 60 mo	IBS
Random survival forest	0.80 ± 0.02	0.80 ± 0.02	0.86 ± 0.04	0.16 ± 0.01
Cox (ridge)	0.80 ± 0.02	0.79 ± 0.02	0.85 ± 0.04	0.16 ± 0.01
CoxNet (Elastic-Net)	0.79 ± 0.02	0.79 ± 0.02	0.84 ± 0.04	0.16 ± 0.01
Component-wise GBS	0.79 ± 0.02	0.79 ± 0.02	0.84 ± 0.04	0.16 ± 0.01
Gradient-boosted survival	0.78 ± 0.03	0.78 ± 0.03	0.84 ± 0.04	0.16 ± 0.02
Extra survival trees	0.78 ± 0.02	0.78 ± 0.02	0.86 ± 0.03	0.17 ± 0.01
Survival SVM (ranking)	0.78 ± 0.03	0.78 ± 0.03	0.84 ± 0.05	—
Milan criteria (reference)	0.68 ± 0.03	—	—	—

**Table 3:** Ablation of the robust multivariate-outlier component and its threshold sensitivity. Part A compares the full pipeline with a variant lacking robust outlier handling. Part B reports the penalised Cox concordance across combinations of the chi-squared significance level (rows) and the robust z-score threshold (columns). Values are Harrell’s concordance index.

Part A. Ablation of Robust Outlier Handling		
Configuration	Harrell C (mean)	SD
Full pipeline (robust MV-outlier imputation)	0.793	0.023
Standard imputation/scaling (no MV handling)	0.795	0.023
Part B —Outlier-threshold sensitivity (Harrell C, penalised Cox)		
χ^2^ significance level	z = 2.5	z = 3.0/3.5
0.950	0.794	0.793/0.793
0.975	0.793	0.793/0.794
0.990	0.794	0.794/0.793

**Table 4:** Hyperparameters selected by the inner cross-validation loop for each learner. The Milan criteria are a fixed clinical rule and require no tuning.

Model	Selected Hyperparameters (Inner Loop)
CoxNet (Elastic-Net)	l1-ratio = 0.5, α = 0.05
Cox (ridge)	α = 100.0
Random survival forest	n estimators = 200, min. samples per leaf = 8
Extra survival trees	n estimators = 300, min. samples per leaf = 8
Gradient-boosted survival	learning rate = 0.05, n estimators = 200, depth = 2
Component-wise GBS	learning rate = 0.1, n estimators = 200
Survival SVM (ranking)	α = 0.5
Milan criteria (reference)	—(fixed clinical rule)

## Data Availability

The data analysed in this study were obtained from the Supplementary Material of the previously published article and can be accessed at: https://www.frontiersin.org/articles/10.3389/fmed.2024.1373005/full#supplementary-material (accessed on 20 March 2026). The Python code used to process these data and to generate the results reported here is available from the corresponding author on reasonable request.

## Supplementary Materials

The following supporting information can be downloaded at: https://www.mdpi.com/article/10.3390/bioengineering13080951/s1, Table S1: Complete hyperparameter search space of the nested cross-validation benchmark. For every learner the table reports the tuned parameters and the exhaustive grid of candidate values, the fixed settings that were not tuned, the number of candidate combinations, the number of inner-loop model fits generated by those combinations, and the configuration selected by the inner loop. The search space is an explicit list of candidate configurations rather than a full factorial product; the search is exhaustive over these candidates, no random, Bayesian or adaptive search was used, and the procedure is fully deterministic under the fixed seed (42).
