# Supplementary material for: A Leakage-Free Survival-Modelling Benchmark for Hepatocellular Carcinoma Recurrence After Liver Transplantation: Nested Cross-Validation Against the Milan Criteria
Source: Bioengineering (Basel). 2026 Aug 21;13(8):951. doi: 10.3390/bioengineering13080951 (PMC13509175; doi:10.3390/bioengineering13080951)
Supplement: Supplementary file 1 [file bioengineering-13-00951-s001.zip › bioengineering-4481342-supplementary.pdf]

bioengineering

MDPI

Supplementary Materials

A Leakage-Free Survival-Modelling Benchmark for Hepatocellular Carcinoma Recurrence After Liver Transplantation: Nested Cross-Validation Against the Milan Criteria

Sami Akbulut [1,2,*], Cemil Colak [2] and Emek Guldogan [2]

[1] Department of Surgery and Liver Transplantation, Inonu University Faculty of Medicine, 44280, Malatya, Türkiye; akbulutsami@gmail.com (SA)
[2] Department of Biostatistics and Medical Informatics, Inonu University Faculty of Medicine, 44280, Malatya, Türkiye; cemilcolak@yahoo.com (CC); emek.guldogan@inonu.edu.tr (EG)
* Correspondence: akbulutsami@gmail.com4 01

Table S1. Complete hyperparameter search space of the nested cross-validation benchmark. For every learner, the table reports the tuned parameters and the exhaustive grid of candidate values, the fixed settings that were not tuned, the number of candidate combinations, the number of inner-loop model fits generated by those combinations, and the configuration selected by the inner loop. The search space is an explicit list of candidate configurations rather than a full factorial product; the search is exhaustive over these candidates, no random, Bayesian or adaptive search was used, and the procedure is fully deterministic under the fixed seed (42).

Learner	Tuned hyperparameters and candidate configurations	Fixed (non-tuned) settings	Candidate combinations	Inner-loop model fits	Value selected by the inner loop
CoxNet (Elastic-Net Cox)	4 explicit candidate configurations: (l1_ratio 0.5, alpha 0.05) (l1_ratio 0.9, alpha 0.05) (l1_ratio 0.5, alpha 0.10) (l1_ratio 0.9, alpha 0.02)	fit_baseline_model = True single alpha per candidate	4	180	l1_ratio = 0.5 alpha = 0.05
Cox (ridge)	alpha: {0.1, 1.0, 10.0, 100.0}	ties = Breslow	4	180	alpha = 100.0
Random survival forest	3 explicit candidates (n_estimators, min_samples_leaf): (200, 8), (200, 16), (400, 8)	max_features = sqrt random_state = 42	3	135	n_estimators = 200 min_samples_leaf = 8
Extra survival trees	2 explicit candidates (n_estimators, min_samples_leaf): (300, 8), (300, 16)	max_features = sqrt random_state = 42	2	90	n_estimators = 300 min_samples_leaf = 8
Gradient-boosted survival trees	3 explicit candidates (learning_rate, n_estimators, max_depth): (0.05, 200, 2), (0.1, 100, 3), (0.05, 300, 2)	loss = coxph random_state = 42	3	135	learning_rate = 0.05 n_estimators = 200 max_depth = 2

Learner	Tuned hyperparameters and candidate configurations	Fixed (non-tuned) settings	Candidate combinations	Inner-loop model fits	Value selected by the inner loop
Component-wise gradient boosting	2 explicit candidates (learning_rate, n_estimators): (0.1, 200), (0.05, 300)	loss = coxph<br>random_state = 42	2	90	learning_rate = 0.1<br>n_estimators = 200
Survival SVM (ranking)	alpha: {0.5, 1.0, 2.0}	max_iter = 200<br>random_state = 42	3	135	alpha = 0.5
Milan criteria (reference)	not applicable — fixed clinical rule	single lesion ≤ 5 cm, or multiple lesions with maximum diameter ≤ 3 cm	0	0	not tuned
**Total (7 tuned learners)**	—	—	**21**	**945**	+ 105 outer-fold refits

Notes. The evaluation design was three repeats of a five-fold outer loop (15 outer folds) with a three-fold inner tuning loop. Every candidate combination was evaluated in every inner fold, so the number of inner-loop model fits for a learner is (candidate combinations) × 3 inner folds × 15 outer folds. Summed across the seven tuned learners this gives 21 × 3 × 15 = 945 inner-loop fits; the selected configuration of each learner was then refitted once per outer fold on the corresponding outer training partition, adding 7 × 15 = 105 refits. Selection within the inner loop used Harrell's concordance index. All data-dependent preprocessing steps (robust Minimum Covariance Determinant outlier screening, cell-wise robust z-score flagging, iterative imputation, log transformation and standardisation) were nested inside the same estimator pipeline and were therefore refitted from scratch for every candidate combination in every inner fold; the fit counts above refer to complete pipeline fits, not to model fits on pre-transformed data. The Milan criteria were applied as a fixed, non-trainable clinical rule in every outer test fold and required no tuning. Parameter names follow the scikit-survival 0.27.0 application programming interface.
